# Healthcare costs attributable to the treatment of patients with spinal metastases: a cohort study with up to 8 years follow-up

**DOI:** 10.1186/s12885-015-1357-z

**Published:** 2015-05-05

**Authors:** Line Stjernholm Tipsmark, Cody Eric Bünger, Miao Wang, Søren Schmidt Morgen, Benny Dahl, Rikke Søgaard

**Affiliations:** 1 Health Economics, CFK - Public Health and Quality Improvement, Central Denmark Region, Olof Palmes Allé 15, 8200 Aarhus N, Denmark; 2 Department of Orthopaedic E, Aarhus University Hospital, Nørrebrogade 44, 8000 Aarhus C, Denmark; 3 Department of Orthopedic Surgery, Spine Unit, Rigshospitalet and University of Copenhagen, Blegdamsvej 9, 2100 København Ø, Denmark; 4 Department of Public Health, Aarhus University, Bartholins Allé 2, 8000 Aarhus C, Denmark; 5 Department of Clinical Medicine, Aarhus University, Palle Juul-Jensens Boulevard 82, 8200 Aarhus N, Denmark

**Keywords:** Healthcare costs, Spinal metastases, Spinal surgery, Survival, Palliative treatment

## Abstract

**Background:**

Cancer treatment, and in particular end-of-life treatment, is associated with substantial healthcare costs. The purpose of this study was to analyse healthcare costs attributable to the treatment of patients with spinal metastases.

**Methods:**

The study population (n = 629) was identified from clinical databases in Denmark. Patients undergoing spinal metastasis treatment from January 2005 through June 2012 were included. Clinical data were merged with national register data on healthcare resource use, costs and death date. The analytic period ranged from treatment initiation until death or administrative censoring in October 2013. Analysis of both survival and costs were stratified into four treatment regimens of increasing invasiveness: radiotherapy (T1), decompression (T2), decompression + instrumentation (T3) and decompression + instrumentation + reconstruction (T4). Survival was analysed using Kaplan-Meier curves. Costs were estimated from a healthcare perspective. Lifetime costs were defined as accumulated costs from treatment initiation until death. The Kaplan-Meier Sampling Average method was used to estimate these costs; 95% CIs were estimated using nonparametric bootstrapping.

**Results:**

Mean age of the study population was 65.2 years (range: 19-95). During a mean follow-up period of 9.2 months (range: 0.1-94.5 months), post treatment survival ranged from 4.4 months (95% CI 2.5-7.5) in the T1 group to 8.7 months (95% CI 6.7-14.1) in the T4 group. Inpatient hospitalisation accounted for 65% and outpatient services for 31% of the healthcare costs followed by hospice placements 3% and primary care 1%. Lifetime healthcare costs accounted for €36,616 (95% CI 33,835-39,583) per T1 patients, €49,632 (95% CI 42,287-57,767) per T2 patient, €70997 (95% CI 62,244-82,354) per T3 patient and €87,814 (95% CI 76,638-101,528) per T4 patient. Overall, 45% of costs were utilised within the first month. T1 and T4 patients had almost identical distributions of costs: inpatient hospitalisation averaged 59% and 36% for outpatient services. Costs of T2 and T3 were very similarly distributed with an average of 71% for inpatient hospitalisation and 25% for outpatient services.

**Conclusion:**

The index treatment accounts for almost half of lifetime health care costs from treatment initiation until death. As expected, lifetime healthcare costs are positively association with invasiveness of treatment.

## Background

Cancer patients are frequently affected by bone metastases and approximately fifty percent of bone metastases are located in the spine [1]. Symptomatic spinal metastases often have severe negative effects on the quality of life due to pain and neurologic dysfunction [2,3]. Metastases can result in epidural spinal cord compression which may cause permanent paraplegia if not treated within 24–48 hours after onset of symptoms [3,4]. When tumours metastasise to bone, the condition is most often incurable and the patients usually have a relatively short life expectancy [5,6]. The choice of optimal treatment therefore should be based on whether the expected outcome outweighs the disutility and risk to the patient of undergoing the treatment [7]. In clinical practice, this choice is often guided by assessment of predicted survival based on prognostic scoring systems such as the Tokuhashi score [8-10]. In this scoring system, patients are scored from 0-15 and a score of 0-8 indicates a predicted survival of 0-6 months, 9-11 predicts 6-12 months survival and 12-15 predicts a survival >12 months. On the basis of the predicted survival different treatment modalities are recommended: conservative treatment, palliative surgery or excisional procedures [8].

In Denmark, cancer treatment accounts for approximately 8% of the total costs of hospital activity [11]. A substantial share of these costs concern end-of-life treatment. Approval of expensive end-of-life treatment has been increasingly debated in the last years and a supplement to the National Institute for Health and Care Excellence guidelines on technology appraisal was written to allow approval of very restricted end-of-life medicines exceeding conventional threshold levels of £20-30,000 per quality-adjusted life years (QALY) [12]. Presently, the evidence for healthcare costs of patients with bone metastases is limited [13-15]. Additionally, lifetime costs attributable to the treatment of patients with spinal metastases have to our knowledge never been analysed [16]. To support decision-making in the field of spinal metastasis treatment, it seems relevant to analyse these costs. The purpose of the present study thus was to analyse healthcare costs attributable to the treatment of patients with spinal metastases from a healthcare perspective.

## Methods

### Study population

The study population consisted of patients with acute symptoms of metastatic epidural spinal cord compression. The diagnosis was based on Magnetic Resonance Imaging combined with clinical symptoms of back pain and/or neurologic impairment. Patients treated from 2005 and onward were included to ensure that patients were representative regarding modern oncological treatment strategy and due to the fact that national data on service costs were unavailable for the period prior to this year. Spinal metastasis patients were identified using two clinical databases Aarhus Spinal Metastases Database and Copenhagen Spinal Metastases Database. Together they represent tertiary referral units serving almost 4 million people, corresponding to 68% of the Danish population. Copenhagen Spinal Metastases Database includes all patients treated with radiotherapy. Aarhus spinal Metastases database includes all spinal metastases patients referred to surgical treatment. From Aarhus Spinal Metastases Database we included 210 consecutive surgical patients over a period of seven years from January 2005 until June 2012. From Copenhagen Spinal Metastases Database we included 419 patients that had been consecutively included in 2011; all treated with radiotherapy. The databases were never meant to be identical and they are the only available spinal metastases databases in Denmark (covering two different geographical areas). Date of treatment initiation (day of surgery or first day of radiation therapy) was recorded and patients were followed until death or administrative censoring in October 2013. The following patient information was extracted from the databases: patient ID, age, gender, diagnosis, Tokuhashi score, procedure codes and day of treatment initiation.

The study was approved by The Danish Data Protection Agency (J.no. 2007-58-0010). Ethical approval from The National Committee on Health Research Ethics was not necessary, since this is not a clinical trial involving biological material or human subjects. For the same reason no written consent was necessary.

### Treatment regimens

Radiotherapy consisted of a short-course regime in patients with expected survival less than 6 months. They received 5 x 4 Gy in one week. Patients with better prognosis underwent 20 x 2 Gy over a period of four weeks. Radiotherapy was administered to the involved vertebra after CT-based three-dimensional planning. The radiotherapeutic regime is hereafter referred to as T1.

Surgical interventions included three main entities with increasing invasiveness: decompression (T2), decompression + instrumentation (T3) and decompression + instrumentation + reconstruction (T4). The spine surgeons in Aarhus divided the worst prognosis group (0 – 8 points) of the Tokuhashi scoring system into two subgroups: patients in the first treatment regime (0 – 4 points) had a life expectancy of less than 3 months. Patients in the second treatment regime (5 – 8 points) were expected to survive between 3 and 6 months. A simple decompression/laminectomy surgery was designed for spinal metastasis patients with the lowest Tokuhashi score (0 – 4 points). Decompression + pedicle screw system instrumentation implantation surgery was employed for patients with low Tokuhashi scores (5 – 8 points). The pedicle screw system strengthens the stability of the spinal segments with metastases. The final surgical regime for patients with an expected survival of over 6 months (9 – 15 points) was posterior decompression with pedicle screw instrumentation and anterior/posterior reconstruction by bone cementing and/or bone graft transplantation. Spine surgeons needed to remove tumour tissues both inside the spinal canal and in the vertebral body followed by reconstruction afterwards. These procedures provide strong mechanical supports and stability of the patients’ spines. The patients were stratified according to the treatment they received (T1-T4).

### Healthcare costs

Lifetime healthcare costs were defined as accumulated costs from treatment initiation until death. The cost perspective of the analysis was healthcare. Primary sector visits were divided by type of care: general practitioner, medical specialist, therapists and other. For hospital sector, inpatient admissions and bed days were recorded as well as outpatient visits. The healthcare costs were retrieved through the National Health Insurance Service Register and The Danish National Patient Register and they were based on Diagnosis-Related Groups, Danish Ambulant Grouping System and collectively bargained (primary sector) tariffs. Patients’ hospice use rates before 2009 were assumed to follow rates from 2009 and onwards, as the former were not available in registries. All costs were converted to price year 2012 by use of the general consumer price index. The healthcare costs were converted to Euros at an exchange rate of 7.45 DKK/€.

### Analyses

Survival was calculated in days from the date of treatment initiation to death or censoring. Cox regression was used to estimate survival. Kaplan-Meier survival curves were performed for each treatment subgroup. The Kaplan-Meier Sampling Average method was used to calculate expected lifetime healthcare costs in order to handle bias introduced by censoring [17]. We partitioned the follow-up period into monthly intervals and hereafter a three-step procedure was followed: 1. Calculating probability of survival at the beginning of each monthly interval, 2. Calculating monthly average costs for patients alive at the beginning of the time interval, 3. Multiplying estimates from step 1 and 2. Hereafter costs were accumulated for each treatment regime. Ninety-five percent confidence intervals (95% CIs) were estimated for probabilities, resource use and costs (calculated separately in step 1 and 2) by use of 1000 nonparametric bootstrap replications.

All statistical analyses were carried out in Stata 13.0 (Stata Corporation, Texas, USA). Tables and graphs were illustrated by use of Excel 2007 (Microsoft Corporation, USA).

## Results

### Patient characteristics

The study population (n = 629) consisted of 60.3% males and the mean age was 65.2 years (range: 19-95). Patients were followed for a mean period of 9.2 months (range: 0.1-94.5 months). The overall censoring rate was 0.09. The censoring rates for the different treatment regimes were 0.11 (T1), 0.03 (T2), 0.11 (T3) and 0.01 (T4). Table 1 shows baseline characteristics for each database.

**Table 1:** **Baseline characteristics of 629 spinal metastases patients**

	Copenhagen database (n = 419)		Aarhus database (n = 210)	
	n	%	n	%
Male	245	58	134	64
Age (years)				
15-54	62	15	42	20
55-64	115	27	68	32
65-74	137	33	71	34
75-84	81	19	26	12
≥85	24	6	3	1
Primary cancer				
Prostate	97	23	67	32
Lung	99	24	22	10
Breast	75	18	41	20
Other	148	35	80	38
Predicted survival time* (months)				
0-6	198	47	107	51
6-12	122	29	76	36
>12	99	24	27	13
Treatment				
T1	419	100		
T2			51	24
T3			79	38
T4			80	38

*Based on the Tokuhashi scoring system at baseline.

T1 conservative, T2 decompression, T3 decompression + instrumentation, T4 decompression + instrumentation + reconstruction.

### Survival

**Figure 1:**
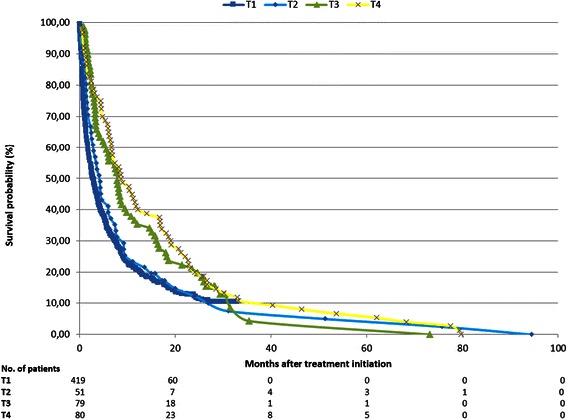
Kaplan-Meier survival curves for each of the four treatment regimes: conservative treatment (T1), decompression (T2), decompression + instrumentation (T3) and decompression + instrumentation + reconstruction (T4).

Figure 1 illustrates the Kaplan-Meier survival curves for the patients in each treatment regime. The number of remaining patients during the follow-up period is shown under the graph. The survival among T1 and T2 patients was very similar until around 25 months where the curve for T1 patients stagnates and T2 proceeds to decrease until 94 months, which was the longest observed follow-up period. T1 patients were observed to have the shortest median survival of 3.0 months (95% CI 2.4-3.8). The median survival time for T2 and T3 patients was longer than preoperatively predicted. T2 patients had a median survival of 4.4 months (95% CI 2.5-7.5) and T3 patients presented with a median survival of 8.0 months (95% CI 5.2-9.9). T4 patients that received the most invasive procedure had the longest median survival of 8.7 months (95% CI 6.7-14.1). Fifty-nine patients were alive at the time of censoring.

### Healthcare resource use

Healthcare resource use categorised by healthcare sector and treatment strategy is shown in Table 2. Evaluating the overall distribution of healthcare resource use, approximately 58% was utilised within the primary sector; furthermore treatment regimes with shorter survival had lower healthcare resource use. Generally, a large number of hospital bed days were observed. Comparing treatment regimes, T1 patients had the highest share of resource use at 38% in outpatient settings and the lowest share in the primary sector at 48%. Resource use among T1 patients was almost evenly distributed between primary and hospital sector. The distribution of resources among T2-T4 patients was more similar, with the majority situated in the hospital sector. Sixty-four percent of T2 resources were used in primary sector and 11% at inpatient admissions, which was the highest percentage among the treatment regimes. T4 patients generally used more resources in the hospital sector compared to the other surgical treatments (T2 and T3).

**Table 2:** **Total healthcare resource use per spinal metastases patient from treatment initiation until death**

	T1 (n = 419)		T2 (n = 51)		T3 (n = 79)		T4 (n = 80)	
	Mean	CI	Mean	CI	Mean	CI	Mean	CI
Primary sector (n)								
GP	14.5	(13.6-15.4)	30.8	(27.0-35.2)	36.0	(33.2-39.2)	34.1	(31.2-37.4)
MS	0.6	(0.5-0.8)	1.5	(1.0-2.1)	1.3	(0.8-2.0)	0.8	(0.6-1.2)
Therapists	4.7	(3.8-5.7)	6.6	(3.7-10.5)	8.6	(5.6-12.1)	9.7	(7.0-12.5)
Other	0.2	(0.2-0.3)	0.4	(0.2-0.6)	0.34	(0.2-0.5)	0.7	(0.5-0.9)
Hospital sector (n)								
Outpatient	16.1	(14.4-17.9)	14.3	(10.8-18.0)	18.7	(14.9-23.7)	24.7	(20.0-29.8)
ER	1.2	(1.1-1.3)	0.7	(0.4-0.9)	0.3	(0.2-0.5)	0.3	(0.2-0.4)
Inpatient								
Admissions	4.4	(4.1-4.7)	6.9	(6.0-7.9)	7.2	(6.4-7.9)	7.9	(7.0-8.8)
Bed days	25.4	(22.9-27.9)	39.2	(32.0-46.9)	45.6	(38.4-54.0)	46.1	(39.4-53.1)
Hospice (n)								
Admissions	0.2	(0.2-0.3)	0.2	(0.0-0.4)	0.2	(0.1-0.4)	0.4	(0.2-0.6)
Bed days	4.1	(2.9-5.5)	1.7	(0.1-5.5)	5.3	(1.9-10.2)	11.9	(4.8-22.5)

ER Emergency room, GP general practitioner, MS medical specialist, T1 conservative, T2 decompression, T3 decompression + instrumentation T4 decompression + instrumentation + reconstruction.

Table 3 summarises the lifetime healthcare costs for the different treatment regimes. The main cost driver was hospital sector accounting for 96% of total costs shared among inpatient hospitalisation at 65% and outpatient visits at 31%, these were followed by hospice accounting for 3% and 1% for primary sector. Overall, 45% of healthcare costs were utilised within the first month after treatment initiation and 90% were utilised after 20 months.

**Table 3:** **Total healthcare cost per spinal metastases patient from treatment initiation until death or censoring**

	T1 (n = 419)			T2 (n = 51)			T3 (n = 79)			T4 (n = 80)		
	Mean	CI	%	Mean	CI	%	Mean	CI	%	Mean	CI	%
Primary sector (€)												
GP	247	(228-266)	1	648	(542-773)	1	647	(580-716)	1	608	(535-682)	1
MS	28	(19-39)	0	46	(29-64)	0	64	(24-122)	0	37	(22-57)	0
Therapists	85	(69-104)	0	92	(46-152)	0	145	(100-198)	0	205	(151-265)	0
Other	32	(26-38)	0	43	(29-60)	0	37	(25-49)	0	62	(50-77)	0
Hospital sector (€)												
Outpatient	13567	(11585-16104)	37	11830	(7124-18411)	24	19005	(12259-28984)	27	31408	(21797-44086)	36
ER	146	(135-157)	0	93	(63-131)	0	40	(24-59)	0	36	(20-57)	0
Inpatient	21421	(19651-23232)	59	36202	(30944-42415)	73	49651	(45007-54859)	70	52,135	(47398-57464)	59
Hospice (€)	1089	(763-1509)	3	678	(81-1572)	1	1409	(559-2631)	2	3324	(1348-6027)	4
**Total**	**36616**	**(33835-39583)**	**100**	**49632**	**(42287-57767)**	**100**	**70997**	**(62244-82354)**	**100**	**87814**	**(76638-101528)**	**100**

ER Emergency room, GP general practitioner, MS medical specialist, T1 conservative, T2 decompression, T3 decompression + instrumentation T4 decompression + instrumentation + reconstruction.

T1 and T4 patients had almost identical distribution of costs: inpatient hospitalisation averaged 59% and outpatient services accounted for 37% and 36%. T1 costs for hospice placements averaged 3% and 4% for T4. Likewise, T2 and T3 costs were almost evenly distributed among sectors with 73% and 70% for inpatient hospitalisation, 24% and 27% for outpatient services. Hospice placements accounted for 1% (T2) and 2% (T3).

**Figure 2:**
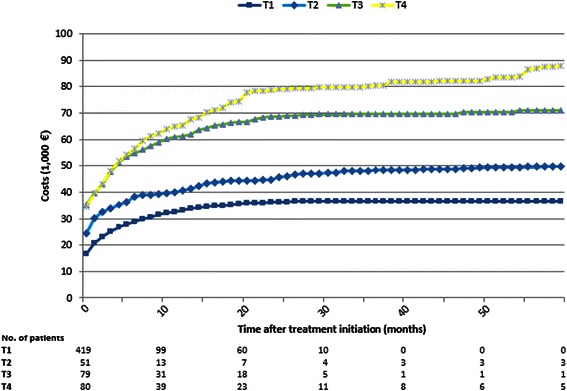
Lifetime healthcare costs per patient for four different treatment regimes: conservative treatment (T1), decompression (T2), decompression + instrumentation (T3) and decompression + instrumentation + reconstruction (T4).

Lifetime costs of the treatment regimes are illustrated in Figure 2. The curves start at the initial treatment cost and increases with resource use hereafter. The cost for T1 patients converged to its maximum relatively quickly and was stable after 36 months due to no survivors. Costs for T1 and T2 patients were very similar except for an upwards shift of the T2 patients due to the extra initial treatment costs and a slightly higher cost rate in the mid-period of follow-up due to longer survival. For the most invasive surgical procedures (T3 and T4) it was seen that within the first 4-5 months after treatment, the healthcare costs were almost identical. Hereafter, the T4 patients demonstrate a continued healthcare resource use whereas the T3 patients’ healthcare use almost levelled out after 30 months due to limited survival time.

## Discussion

To our knowledge, this is the first study analysing healthcare costs attributable to the treatment of patients with spinal metastases. The lifetime cost of healthcare after being referred to specialised treatment for spinal metastases range from €36,616 (95% CI €33,835-39,583) to €87,814 (95% CI €76,638-101,528) per patient, depending on the type of index treatment received. As expected it was found that the main category driver was inpatient hospitalisation, accounting for about 65% of total costs, followed by outpatient visits, accounting for another 31%. Also as expected, the ranking of lifetime costs was in accordance with the observed survival of the four treatment regimes: longer survival was associated with higher healthcare costs.

In the field of spinal metastasis research, the present study population is unique in size and follow-up period because a part of the study population originates from one of the oldest and most extensive spinal metastases databases in the world [10]. Although the study population represents one of the largest samples in the literature, we do not have statistical power to assess the role of patient characteristics in a multivariate analysis. A strength of this study is that the clinical data is merged with unique register data, which does not contain missing data and is not affected by recall bias. On the weakness side, one could consider whether the applied DRG tariffs reflect the actual economic opportunity cost. In general, DRG tariffs are national average costs of related procedures and the number of procedures does therefore not match the number of available tariffs.

Since the present study is the first of its kind, comparison is difficult. A study published by Vera-Llonch et al. describes healthcare costs in women with metastatic breast cancer receiving chemotherapy [15]. Methodically the studies are comparable; both apply the Kaplan-Meier Sampling Average to calculate total costs. Our study, however, possess substantial additional strength by reporting bootstrapped confidence intervals for both costs and survival as well as providing a description of the treatment regimes administered.

The results of Vera-Llonch et al.’s study can be compared to the results of T1 patients, since both groups receive non-surgical treatment, but certain reservations have to made since the diagnoses among these groups are not entirely the same (in our study breast cancer patients only comprised 18.1% of the T1 treatment regime). The total costs per breast cancer patient in Vera-Llonch et al.’s study was estimated to be $128,556 (≈ €94,000), which is more than twice the costs of T1 patients (€36,616) in our study. Breast cancer patients are generally observed to have longer survival compared to patients with most other cancer forms [8,10]. In Vera-Llonch et al.’s study this results in a follow-up period twice as long as ours. During this period, a larger proportion of the study population is alive and thereby able to consume healthcare resources compared to our study population. This could be a possible explanation for the higher costs, together with the fact that Vera-Llonch et al. also include outpatient medication costs. Comparing the healthcare utilisation of the present study with utilisation reported in Vera-Llonch et al.’s study for breast cancer patients, it appears that Danish patients in the follow-up period use more health care in the hospital sector: hospital admissions (1.7 versus 4.4 admissions), and inpatient days (10.7 versus 25.4 days per remainder life time) and substantially less in the primary sector: outpatient services (83.6 versus 30.6 visits per follow-up). These differences however could be explained from differences in study designs, as Vera-Llonch et al.’s study included only patients with breast cancer who might have been on average less severe than patients in the present study. Furthermore, the above-mentioned differences in follow-up period could be part of the explanation.

The present study was conducted from a healthcare perspective so one must consider omitted costs (informal care giving by relatives, transportation back and forth from the hospital, terminal care in the patient’s own home and over-the-counter medication) if results are to be compared to societal costs of treating patients with spinal metastases. A potential limitation of the study could be a low degree of external validity since the organisation of the treatment of patients with spinal metastases in Denmark is centralised compared to other countries. This centralisation, however, makes the study population representative. Furthermore, considerations must also be given to differences in price standards between countries.

Since this analysis does not compare treatment costs and effects, these results cannot be used to conclude on the cost-effectiveness of the treatments. Guided by predicted survival, patients were carefully selected for a specific treatment regime and this is one of the reasons for T1 patients having the shortest survival and likewise the lowest cost etc.

## Conclusion

We believe that the results of the present study can be used to inform the cost side of future cost effectiveness analyses, thereby supporting decision making about costly end-of-life treatment. The results of such a cost effectiveness analysis would contribute to optimising the basis of decision-making. Future research of societal perspective would be of great interest as well as investigating quality of life parameters among this patient group.

The index treatment accounts for almost half of lifetime health care costs from treatment initiation until death followed by readmissions and outpatient visits. Hospice placements and primary care account for only a minor share of total costs. As expected, lifetime healthcare costs are positively association with invasiveness of treatment.
